# Screening for Cardiac Amyloidosis When Conducting Carpal Tunnel Surgery

**DOI:** 10.3390/jcm14113710

**Published:** 2025-05-26

**Authors:** Sofia Pimenta, Luís Santos, Ana Martins, Janete Santos, Inês Fortuna, Barbara Pereira, Mariana Vasconcelos, Miguel Carvalho, André Carvalho, Micaela Gonçalves, Isabel Pinto, Isabel Fidalgo, Jorge Pereira, Teresa Faria, Lúcia Costa, Elisabete Martins

**Affiliations:** 1 Department of Rheumatology, Centro Hospitalar Universitário de São João, E.P.E., 4200-319 Porto, Portugal; sofiadsp@sapo.pt (S.P.); anaigmartins.med@gmail.com (A.M.); isabelfidalgo1968@gmail.com (I.F.); dias.costa.ml@gmail.com (L.C.); 2 Department of Medicine, Faculty of Medicine, University of Porto, 4200-319 Porto, Portugal; ebernardes@med.up.pt (E.M.); inesfortuna@gmail.com (I.F.); 3 Department of Cardiology, Centro Hospitalar Universitário de São João, E.P.E., 4200-319 Porto, Portugal; luis_santos1345@hotmail.com (L.S.); mv.mvmar@gmail.com (M.V.); jmiguelmartinscarvalho@gmail.com (M.C.); 4 Nuclear Medicine Department, Centro Hospitalar Universitário de São João, E.P.E., 4200-319 Porto, Portugalandre@med.up.pt (A.C.); jorgepgpereira@gmail.com (J.P.); mteresafaria@gmail.com (T.F.); 5 Department of Orthopaedics, Centro Hospitalar Universitário de São João, E.P.E., 4200-319 Porto, Portugal; claudiamicaela.fg@gmail.com (M.G.); isabelmcap@gmail.com (I.P.)

**Keywords:** carpal tunnel syndrome, cardiac amyloidosis, cardiac scintigraphy

## Abstract

**Background**: Carpal tunnel syndrome (CTS) has emerged as an early indicator of cardiac amyloidosis (CA) caused by transthyretin-associated (ATTR) mutations, possibly linked to adverse cardiovascular outcomes. This case series examines the relationship between idiopathic CTS and CA imaging diagnosis. **Methods**: Twenty-two patients from the cross-sectional study CarPoS (NCT05409833) were included. These patients underwent physical evaluation, laboratory exams, electrocardiography, echocardiography, cardiac magnetic resonance (CMR) imaging, and scintigraphy with ^99m^Tc-3,3-diphosphono-1,2-propanodicarboxylic acid. **Results**: Four of the twenty-two patients included had ATTR cardiomyopathy. These patients presented left-ventricle hypertrophy and signs of infiltrative cardiomyopathy in echocardiograms and late gadolinium enhancement in CMR images without having any cardiovascular symptoms. **Conclusions**: Our findings suggest a high prevalence of CA in patients with bilateral idiopathic CTS, highlighting the importance of screening for CA in patients with CTS. Early detection could significantly impact patient prognosis, underscoring the need for further research into diagnostic and therapeutic strategies.

## 1. Introduction

Transthyretin-associated (ATTR) amyloidosis is one of the leading causes of cardiac amyloidosis (CA), a condition whose occurrence is due to either genetic ATTR mutations or the misfolding of the wild-type transthyretin (ATTRwt) protein. This systemic disorder, which is primarily age-related, manifests in various organs, including the heart, often preceding a CA diagnosis. Carpal tunnel syndrome (CTS) has emerged as an early indicator that is potentially linked to adverse cardiovascular outcomes [1,2,3,4]. A recent study on 538 patients identified CTS as an independent risk factor with an increased risk of death, especially among ATTRwt patients (HR 3.63, 95% CI [1.27–10.3]) [5].

In the study by Sperry et al., patients who underwent open carpal release surgery for idiopathic CTS (20%) had previously unknown cardiac involvement [4]. A one-year delay in diagnosis was associated with an increase in natriuretic peptide levels, cardiac conduction disturbances, and atrial fibrillation (AF). There is a significant delay in the diagnosis of CA in ATTR patients, and this delay is negatively associated with access to treatment and long-term survival. A Danish registry found that CTS increased the rate of amyloidosis diagnosis 12-fold, although the absolute risk was low [6]. In the same registry, patients with heart failure (HF) and CTS had greater long-term mortality, possibly due to the development of ATTRwt CA, suggesting that CTS could be an early marker for adverse cardiovascular outcomes [4].

With advances in ATTR CA treatment, early diagnosis could significantly improve the prognosis. Cardiac scintigraphy with bone markers allows noninvasive cardiac imaging with high specificity and sensibility for the diagnosis of ATTR CA, allowing the early screening of CA for patients with idiopathic CTS using the Perugini semi-quantitative grading score of cardiac uptake following the injection of radiotracers.

The CarPoS is a cross-sectional clinical study (trial registration number: NCT05409833) excluding patients with specific comorbidities. It has received ethical approval (reference No: CE 200/2020) and, as such, adheres to established guidelines. The preliminary findings based on our cohort underscore the relevance of exploring this association further.

The connection between CTS and CA, particularly ATTR CA, arose from the observation that amyloid, predominantly transthyretin, can deposit in the tenosynovial tissues and ligaments within the carpal tunnel, potentially causing median nerve compression and the development of CTS [7,8,9]. Importantly, CTS, especially when bilateral, is increasingly recognized as a potential early indicator, or “red flag”, for systemic amyloidosis, which can subsequently involve the heart [3]. This extracardiac manifestation often precedes the onset of overt cardiac symptoms by several years, presenting a potential window for early diagnosis and intervention [3]. Studies have also reported a significant prevalence of CTS in patients already diagnosed with cardiac amyloidosis [2]. Given this association and the therapeutic advancements in managing CA, particularly ATTR CA, in this report, we aim to evaluate the current research to determine the evidence supporting routine screening for CA when conducting CTS surgery.

## 2. Materials and Methods

CarPoS (NCT05409833) is a cross-sectional clinical study investigating the relationship between idiopathic CTS and ATTR amyloidosis. This case report features consecutive patients aged ≥60 years old with symptomatic CTS from the CarPoS study who were recruited after attending an orthopedics consultation; identified as requiring hand surgery at the university tertiary hospital, Centro Hospitalar Universitário de São João (CHUSJ); and referred to a rheumatology consultation. Toarthritis study only idiopathic CTS, patients with diabetes mellitus, hypothyroidism, chronic renal failure under hemodialysis, rheumatoid arthritis, and other inflammatory arthropathies, multiple myeloma, gout, chondrocalcinosis, Colles’s fracture, space-occupying lesions, or infectious synovitis were excluded. CTS was diagnosed based on each patient’s clinical history, a physical examination, nerve conduction studies, and a wrist ultrasound. Physical examinations included the Tinel sign at the wrist, the Phalen test, and sensory disturbance detected over the median nerve-innervated area. The rheumatologist conducted an ultrasound of the wristThe rheumatologist rheumatologist conducted an ultrasound of the wrist to measure the transversal area of the median nerve and confirm the absence of space-occupying lesions or anatomical abnormalities.

Patients also underwent other comprehensive clinical evaluations, including laboratory tests, electrocardiography, echocardiography, cardiac magnetic resonance (CMR) imaging, and scintigraphy with ^99m^Tc-3,3-diphosphono-1,2-propanodicarboxylic acid (^99m^Tc-DPD).

This project was approved by the Ethics Committee for Health of CHUSJ (reference No: CE 200/2020) according to the principles of the Helsinki Declaration, the Convention on Human Rights and Biomedicine, and the guidelines of the Council for International Organizations of Medical Sciences, and written informed consent was obtained from each patient.

## 3. Results

**Figure 1:**
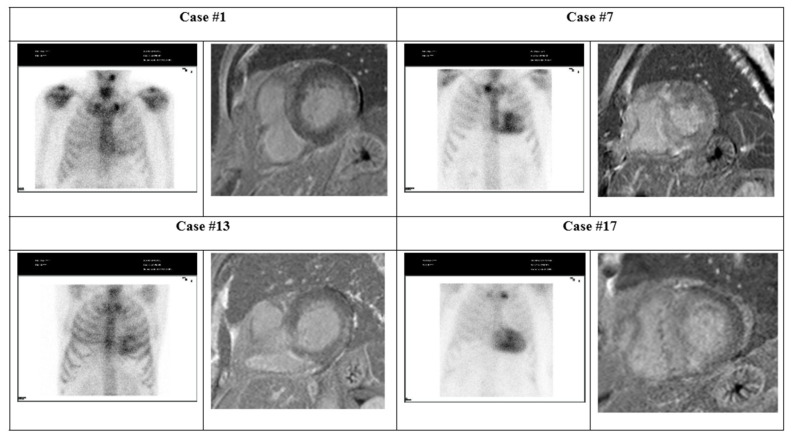
[Cardiac Imaging Evaluation] Patients underwent scintigraphy with 99m Tc-DPD (left) before surgery. Late gadolinium enhancement discernible from cardiac magnetic resonance imaging is evident in the images on the right.

Twenty-two patients were included, of which eight were men, who were between 60 and 86 years old, with a mean age of 72. Most patients had moderate to severe bilateral CTS and did not exhibit cardiac symptoms. The pertinent demographic characteristics and cardiovascular comorbidities are described in Table 1. Four patients had cardiac scintigraphy with high-grade cardiac capture of Perugini grades 2 and 3 (Figure 1).

Table 2 describes the cardiac characteristics of the four cases with positive Perugini grades. All of these patients were male and had hypertension and dyslipidemia. They presented left-ventricular hypertrophy (LVH) with a maximum myocardial thickness ranging from 13 to 21 mm, with an infiltrative appearance (Table 2). These four patients had preserved biventricular systolic function and late gadolinium enhancement (LGE) observable via CMR, with two exhibiting a pattern suggestive of amyloidosis (Figure 1).

In Case #1, the patient presented a pseudo infarction pattern in their electrocardiogram (ECG); the echocardiogram revealed hypertrophy, normal left-ventricular (LV) global longitudinal strain (GLS), and basal lateral intra-myocardial LGE determined via CMR.

In Case #7, the echocardiogram revealed hypertrophy and diminished GLS. CMR images showed diffuse intra-myocardial LGE, slightly more pronounced in the interventricular septum.

In case #13, the ECG revealed sinus bradycardia. The echocardiogram revealed hypertrophy and a standard GLS, and the CMR showed basal inferolateral intra-myocardial LGE.

In Case #17, the ECG revealed a pseudo-infarction pattern. The echocardiogram revealed a diminished GLS, and CMR showed subendocardial LGE in the basal segments and diffused intra-myocardial in the mid-apical septal segments.

## 4. Discussion

In our study, 4 of the 22 patients had ATTRwt cardiomyopathy. Although these results are still preliminary and were obtained from a small sample, the patients with bilateral idiopathic CTS seemed to have a high prevalence of CA. This study highlights the importance of screening for CA in patients with CTS, especially patients aged 60 years or older with bilateral idiopathic CTS.

The etiology of CTS remains unclear in most cases. The prevalence of amyloid deposition in patients with CTS has been investigated in some studies [6,7,8], and there is evidence showing that CTS is one of the most common and early clinical manifestations of senile systemic amyloidosis and should be considered a red-flag symptom [7,9,10].

Numerous investigations focusing on scintigraphy using bone-seeker agents like ^99m^Tc-DPD have proven they offer high sensibility and specificity in diagnosing CA [11,12,13,14,15]. Cardiac scintigraphy with bone-avid radiotracers proved to be particularly sensitive in the early identification of ATTR CA, including in ATTR mutation carriers without apparent cardiac involvement diagnosed using other diagnostic techniques [14,15,16]. Cardiac scintigraphy is appropriate for screening for CA in CTS patients. However, it is not clear which CTS patients should undergo this exam and whether the exam can be preceded or replaced by other imaging methods, such as echocardiography.

### 4.1. The Role of Advanced Echocardiography in the Diagnosis of Cardiac Amyloidosis

Standard echocardiography can reveal several findings suggestive of CA. These include an unexplained increase in LV wall thickness, often with a preserved ejection fraction [17]. Advanced echocardiographic techniques, particularly speckle-tracking echocardiography and strain imaging have significantly improved the non-invasive diagnosis of CA. A characteristic finding in CA is the “apical sparing” pattern of longitudinal strain, wherein the apical segments of the LV show relatively preserved strain compared to the more severely affected basal and mid-ventricular segments [18,19]. This pattern helps differentiate CA from other causes of LVH, such as hypertension or hypertrophic cardiomyopathy (HCM). Tissue Doppler imaging is another valuable echocardiographic technique that assesses regional myocardial velocities, allowing for detecting early diastolic dysfunction. The “5–5–5” sign, characterized by systolic (s’) and early (e’) and late (a’) diastolic velocities that are all less than 5 cm/s at the mitral annulus, is highly suggestive of advanced CA [20].

All our CTS patients with a Perugini grade of 2 or 3 presented LVH and signs of infiltrative cardiomyopathy in echocardiograms when their neuropathy symptoms were evaluated. Therefore, echocardiographic evaluation may also be helpful for the initial CA diagnosis for CTS patients with LVH for which other causes have been ruled out. However, it is important to note that in earlier stages, this approach may exhibit lower specificity in distinguishing amyloid from other hypertrophic phenotypes. Therefore, the interpretation of echocardiographic findings should always be correlated with clinical red flags, laboratory tests (including those involving cardiac biomarkers), and other cardiac imaging modalities for a definitive diagnosis.

This evidence also suggests that CTS patients have a longer preclinical phase of CA. More evidence obtained via more extensive studies is required in order to determine the clinical implications of performing echocardiography on patients with idiopathic CTS. It is also necessary to determine the usefulness of the initiation of ATTR amyloidosis therapy for these patients and how it could impact their prognoses. Meanwhile, the diagnosis of asymptomatic ATTR CA in CTS patients implies the beginning of regular, long-term cardiac follow-ups.

### 4.2. Comparison of ECG and Imaging Findings

A notable finding pertaining to CA is the discordance between ECG and echocardiography, wherein low-voltage QRS complexes have been observed despite significant LVH observed via echocardiography [21]. This mismatch can be a crucial diagnostic indication of CA in patients with CTS and cardiac abnormalities.

Although Cases #1 and #7 did not show any ECG abnormalities, echocardiography revealed LVH in both patients, with maximum wall thicknesses of 13 mm and 19 mm in CMR, respectively. In addition, both exhibited intra-myocardial LGE. Notably, all patients demonstrated LGE with heterogeneous distribution patterns.

### 4.3. Cost-Effectiveness and Potential Long-Term Clinical Benefits of Routine Screening

Early diagnosis of CA, particularly ATTR CA, offers the potential for significant long-term clinical benefits. The availability of disease-modifying therapies has been shown to be a promising factor in terms of slowing the progression of ATTR-CA and improving patient outcomes. These treatments are generally more effective when initiated in the earlier stages of this disease. Early intervention can prevent or delay the development of severe HF, reduce the risk of mortality, and improve the overall quality of life for affected individuals [22].

However, the cost-effectiveness of implementing routine CA screening at the time of CTS surgery requires careful consideration. Factors such as the prevalence of CA in the screened population, the cost of screening tests, and the expenses associated with subsequent diagnostic workups and treatment must be weighed against the potential benefits. Targeting screening efforts towards higher-risk subgroups, such as older patients with bilateral idiopathic CTS, particularly men, and those with additional red flags like AF, HF, or LVH, may improve the cost-effectiveness of screening programs.

Despite the small number of cases, our study reveals that patients submitted to CTS may already present signs of ATTR CA accompanied by significant cardiac changes despite exhibiting no cardiac symptoms. These results reinforce the importance of cardiac screening for patients with CTS, who are most often evaluated by individuals with varied medical or surgical specialties. More data is needed to establish the best screening strategy for CA in these patients and the ideal follow-up and therapeutic implications for earlier diagnoses.

## 5. Conclusions and Recommendations

The evidence reviewed in this report indicates there is a significant association between CTS and CA, particularly ATTR CA. Follow-up studies consistently demonstrate an elevated risk of developing CA years after CTS surgery, suggesting that CTS can be an early manifestation of a systemic process.

Given the advancements in therapies for ATTR CA, early diagnosis is crucial for improving patient outcomes. Screening at the time of CTS surgery represents a potential opportunity for early detection. While universal screening may not be definitively cost-effective, a targeted approach focusing on higher-risk subgroups appears warranted.

Based on the available evidence, the following recommendations are proposed:

1. Consider screening older patients (≥60 years) undergoing CTS surgery, particularly those with bilateral idiopathic CTS, for CA. This age group has a higher prevalence of ATTRwt CA, and bilateral CTS is a recognized red flag.
2. Incorporate a thorough assessment of cardiovascular risk factors and symptoms for patients presenting CTS surgery. The presence of additional red flags, such as a history of HF, AF, conduction abnormalities, unexplained LVH detected via prior imaging, or elevated levels of cardiac biomarkers, should raise suspicion for underlying CA.
3. Consider obtaining a tenosynovial biopsy at the time of carpal tunnel release surgery from patients meeting the criteria given above or those for whom there is a strong clinical suspicion for amyloidosis. While the immediate yield of detecting cardiac involvement may be low, amyloid in the carpal tunnel tissue warrants further cardiac evaluation and long-term follow-ups.
4. Implement a screening pathway for patients with amyloid deposits in their carpal tunnel tissue but no initial cardiac involvement. This pathway should include regular cardiac evaluations, including echocardiography and biomarker assessment, to monitor the potential development of cardiac amyloidosis over time.
5. Further research is needed to establish the cost-effectiveness of various screening strategies for CA in the context of CTS surgery. Long-term follow-up studies on patients with localized amyloid in the carpal tunnel and comprehensive cost–benefit analyses are essential to refine screening guidelines.

Multidisciplinary collaboration between orthopedic surgeons, hand surgeons, primary care physicians, rheumatologists, cardiologists, and amyloidosis specialists is crucial in order to optimize the identification and management of patients with CTS who may have or be at risk of developing cardiac amyloidosis. Raising awareness among healthcare professionals about this association is essential for facilitating early diagnosis and improving outcomes for this potentially serious condition.

## 6. Limitations

1. Tenosynovial or surgical specimen biopsies were not performed systematically. This was due to several factors, including the primary focus of the case series on cardiac findings, patient refusal, or the absence of surgical specimens in some cases.
2. Genetic testing was not performed for all patients with positive scintigraphy. Genetic testing was prioritized for patients in whom the presence of amyloid deposits had been histologically confirmed. This approach was employed due to resource constraints and the diagnostic algorithm typically followed at our center.
3. The prevalence of subclinical CA in our case series may have been high, which might reflect a selection bias due to the recruitment of patients with bilateral CTS at a specialized referral center. Patients with bilateral CTS are more likely to be referred for further investigation, potentially enriching our sample with individuals at higher risk for underlying conditions like amyloidosis.
4. This is a pilot case series within the larger CarPoS project, and larger validation studies are ongoing.

## Figures and Tables

**Table 1:** Clinical cardiac characterization of carpal tunnel syndrome patients.

Cases	Gender, Age	Cardiovascular Risk Factors/Disease	Perugini	Severity of CTS
1	M, 79	HBP; dyslipidemia	2	Right: Severe; Left: Severe
2	M, 69	Dyslipidemia	0	Right: Moderate; Left: Severe
3	M, 64	Dyslipidemia; previous smoker; coronary disease	0	Right: Moderate; Left: Moderate
4	F, 63	-	0	Right: NA; Left: Moderate
5	F, 63	HBP	0	Right: Moderate; Left: Moderate
6	F, 75	Dyslipidemia; obesity	0	Right: Mild; Left: Mild
7	M, 74	HBP; dyslipidemia; obesity; coronary disease	3	Right: Severe; Left: Mild
8	F, 83	HBP	0	Right: Severe; Left: Severe
9	M, 67	HBP	0	Right: Severe; Left: Severe
10	F, 62	-	0	Right: Mild; Left: Moderate
11	F, 60	HBP	0	Right: Severe; Left: Moderate
12	F, 84	HBP	0	Right: Severe; Left: Mild
13	M, 86	HBP; dyslipidemia	3	Right: Moderate; Left: Mild
14	F, 81	-	0	Right: Severe; Left: Severe
15	F, 70	HBP	0	Right: Moderate; Left: Moderate
16	F, 72	HBP; obesity	0	Right: Severe; Left: Moderate
17	M, 81	HBP; dyslipidemia; obesity	3	Right: Severe; Left: Severe
18	F, 61	-	0	Right: Mild; Left: Mild
19	F, 85	HBP; stroke	0	Right: Moderate; Left: Severe
20	M, 70	Diabetes mellitus type 2	0	Right: Moderate; Left: Moderate
21	F, 60	-	0	Right: Severe; Left: Severe
22	F, 66	HBP	0	Right: Moderate; Left: Severe

CTS, carpal tunnel syndrome, HBP, high blood pressure.

**Table 2:** Cardiac characterization of cardiac amyloidosis patients diagnosed using the Perugini scale.

Exam	Parameter	Case #1	Case #7	Case #13	Case #17
-	CV symptoms	Asymptomatic	Asymptomatic	Asymptomatic	Tiredness
-	Troponin I (ng/mL)	35.3	37.9	10.5	71.7
Scintigraphy	Perugini grade (0–3)	2	3	3	3
Echocardiogram	LVEF (%)	60.2	60.0	77.4	61.4
	GLS (%)	−19.9	−11.7	−18.7	−10.4
	PW (mm)	12.0	14.1	12.0	13.0
	Changes	Concentric LVH	LVH	LVH	Concentric LVH
	E-wave (cm/s)	10.8	9.5	7.3	-
ECG	Changes	None	None	Left anterior fascicular block	1^st^-degree AV block
	Heart rate (bpm)	76	72	50	88
	QRS (ms)	104	104	102	98
	PR (ms)	167	172	173	270
	QT (ms)	346	387	420	355
Cardiac Magnetic Resonance	LVEF (%)	67	50	58	61
	LVH	Moderate	Severe	Moderate	Severe
	Max. thickness (mm)	13	19	14	21
	LGE	1	1	1	1
	LGE ≥ 3 segments	0	0	0	1
	Conclusions	Fibrosis (probable amyloid infiltration)	Findings suggestive of amyloidosis	Fibrosis (probable amyloid infiltration)	Findings suggestive of amyloidosis

AV, atrioventricular; CV, cardiovascular; ECG, electrocardiogram; GLS, global longitudinal strain; LGE, late gadolinium enhancement; LVEF, left-ventricular ejection fraction; LVH, left-ventricle hypertrophy; PW, posterior wall.

## Data Availability

The data presented in this article are not readily available because they are part of an ongoing study. Requests for access to these data should be made to the corresponding author, Janete Santos, who can be reached at sjanete@med.up.pt.

## Abbreviations

The following abbreviations are used in this manuscript:

AF	Atrial fibrillation
ATTR	Transthyretin-associated amyloidosis
AV	Atrioventricular
CA	Cardiac amyloidosis
CMR	Cardiac magnetic resonance
CTS	Carpal tunnel syndrome
CV	Cardiovascular
ECG	Electrocardiogram
GLS	Global longitudinal strain
HBP	High blood pressure
HCM	Hypertrophic cardiomyopathy
HF	Heart failure
LGE	Late gadolinium enhancement
LV	Left ventricle
LVEF	Left-ventricular ejection fraction
LVH	Left-ventricle hypertrophy
PW	Posterior wall
Tc-DPD	Tc-3,3-diphosphonate-1,2-propanodicarboxylic acid
